# Supplementary material for: SARS-CoV-2 Renal Impairment in Critical Care: An Observational Study of 42 Cases (Kidney COVID)
Source: J Clin Med. 2021 Apr 8;10(8):1571. doi: 10.3390/jcm10081571 (PMC8068224; doi:10.3390/jcm10081571)
Supplement: Supplementary file 1 [file jcm-10-01571-s001.pdf]

Table S1. Complete baseline characteristics stratified by the presence/absence of an acute kidney injury during the ICU stay.

	Overall (n = 42)	Non-AKI (n = 18)	AKI (n = 24)	p-Value
Days between hospitalization and admission in ICU	1.00 (0.00, 2.00)	1.00 (0.00, 2.75)	0.00 (0.00, 2.00)	0.351
Days between first symptoms and admission to ICU	8.00 (7.00, 12.00)	8.00 (7.00, 11.00)	8.00 (6.75, 12.25)	0.890
**Blood type (%)**				0.922
A (%)	14 (37.8)	6 (40.0)	8 (36.4)	
AB (%)	3 (8.1)	1 (6.7)	2 (9.1)	
B (%)	8 (21.6)	4 (26.7)	4 (18.2)	
O (%)	12 (32.4)	4 (26.7)	8 (36.4)	
Rhesus + (%)	31 (83.8)	10 (66.7)	21 (95.5)	0.031
**Comorbidities**				
Chronic obstructive pulmonary disease (%)	2 (4.8)	2 (11.1)	0 (0.0)	0.178
Diabete mellitus (%)	10 (23.8)	4 (22.2)	6 (25.0)	1.000
Chronic high blood pressure (%)	19 (45.2)	8 (44.4)	11 (45.8)	1.000
Tumor (%)	3 (7.1)	2 (11.1)	1 (4.2)	0.567
Chronic kidney disease (%)	7 (16.7)	2 (11.1)	5 (20.8)	0.679
Alcohol dependence (%)	1 (2.4)	0 (0.0)	1 (4.2)	
Smokers (%)	4 (9.5)	1 (5.6)	3 (12.5)	
**Chronic Treatments**				
Non-steroidal anti-inflammatory (%)	2 (4.9)	0 (0.0)	2 (8.7)	
Angiotensin II receptor antagonist (%)	5 (12.5)	1 (5.6)	4 (18.2)	
Beta blocker (%)	9 (22.5)	3 (16.7)	6 (27.3)	
Corticoid (%)	1 (2.4)	1 (5.6)	0 (0.0)	
Conversion enzyme inhibitor (%)	7 (17.5)	3 (16.7)	4 (18.2)	
**On Hospital Admission**				
Serum creatinine on hospital admission ($\mu mol \cdot L^{-1}$)	86.00 (67.75, 106.75)	74.00 (60.25, 93.25)	94.00 (76.25, 134.75)	0.037
**Treatment on ICU Admission**				
Anakinra (%)	2 (4.8)	1 (5.6)	1 (4.2)	1.000
Remdesevir (%)	7 (16.7)	4 (22.2)	3 (12.5)	0.438
Beta-2-mimetic (%)	1 (2.4)	0 (0.0)	1 (4.2)	1.000
Third-generation cephalosporin (%)	33 (78.6)	13 (72.2)	20 (83.3)	0.462
Ivermectine (%)	3 (7.1)	3 (16.7)	0 (0.0)	0.071
Macrolide (%)	32 (76.2)	13 (72.2)	19 (79.2)	0.720
Hydroxychloroquine (%)	5 (11.9)	2 (11.1)	3 (12.5)	1.000
**On ICU Admission**				
Serum creatinine on ICU admission ($\mu mol \cdot L^{-1}$)	76.50 (61.75, 100.75)	74.50 (54.25, 93.50)	82.00 (69.25, 118.50)	0.208
Creatinine clearance by MDRD ($mL \cdot min^{-1} \cdot 1.73^{-1}$)	87.72 (64.71, 117.29)	91.80 (72.38, 135.85)	82.47 (54.53, 102.27)	0.213
GFR < 60$mL \cdot min^{-1}$ by MDRD (%)	10 (23.8)	2 (11.1)	8 (33.3)	0.147
ARC (MDRD ICU admission) (%)	10 (23.8)	6 (33.3)	4 (16.7)	0.281
SAPS II score	43.00 (36.50, 60.25)	38.00 (31.75, 60.50)	47.50 (39.75, 57.25)	0.214
FiO_2 (%)	70.00 (50.00, 100.00)	80.00 (60.00, 100.00)	66.00 (50.00, 95.00)	0.195
PaO_2 (mmHg)	85.00 (70.00, 117.00)	87.00 (68.50, 105.50)	81.00 (71.00, 121.00)	0.844

Values are expressed as median (interquartile ranges), absolute value (percentages); [2]ARC (augmented renal clearance), FiO_2 (Fraction of inspired oxygen), GFR (Glomerular Filtration Rate), ICU (intensive care unit), PaO_2 (partial pressure of oxygen in arterial blood), SAPS (simplified acute physiology score), AKI (acute kidney injury).

Table S2. Biological values within the first week after ICU admission.

	Overall (n = 34)	Non-AKI (n = 15)	AKI (n = 19)	p-Value
**Urine Tests**				
Alpha1-microglobulin (mg·L^{-1})	139.50 (63.75, 261.75)	72.00 (31.50, 189.50)	237.00 (86.00, 280.00)	0.050
Urine IgG (mg·L^{-1})	19.65 (10.17, 47.43)	17.40 (7.10, 22.05)	26.70 (13.00, 76.10)	0.064
Urine microalbumin (g·L^{-1})	0.05 (0.03, 0.16)	0.04 (0.03, 0.08)	0.06 (0.03, 0.28)	0.171
Urine protein (g·L^{-1})	0.63 (0.32, 1.15)	0.44 (0.32, 0.74)	0.98 (0.40, 1.35)	0.083
Urine retinol binding protein (mg·L^{-1})	10.96 (0.00, 51.80)	8.57 (0.00, 28.95)	16.50 (3.65, 56.60)	0.332
Urine creatinine (mmol·L^{-1})	6.40 (5.16, 7.46)	6.77 (5.42, 8.97)	6.11 (5.05, 7.10)	0.305
Urine urea (mmol·L^{-1})	291.10 (186.90, 394.00)	348.80 (204.70, 424.30)	249.40 (165.30, 336.33)	0.039
**Blood Tests**				
Serum creatinine (μmol·L^{-1})	77.00 (53.00, 109.00)	58.00 (49.00, 70.50)	106.00 (64.00, 169.25)	0.002
Serum urea (mmol·L^{-1})	9.50 (6.90, 14.50)	6.10 (5.05, 8.65)	11.90 (9.43, 19.10)	<0.001
CRP (mg·L^{-1})	176.20 (78.38, 283.00)	171.40 (78.00, 198.40)	227.60 (80.90, 288.80)	0.420
Ferritine (μg·L^{-1})	1109.00 (678.00, 1861.50)	1050.50 (671.25, 2008.00)	1467.00 (678.00, 1708.50)	0.845
Fibrinogen (g·L^{-1})	8.40 (6.43, 9.57)	8.54 (5.90, 9.42)	7.62 (6.96, 9.56)	0.980
Leucocytes (10^9·L^{-1})	9.05 (7.64, 1.18)	8.78 (7.73, 9.86)	10.6 (7.89, 13.71)	0.182
Interleukin 1 beta (pg·L^{-1})	0.00 (0.00, 0.00)	0.00 (0.00, 0.00)	0.00 (0.00, 0.00)	NA
Interleukin 6 (pg·L^{-1})	98.80 (61.95, 356.30)	69.80 (54.20, 314.70)	123.40 (86.15, 328.15)	0.278
Interleukin 10 (pg·L^{-1})	21.60 (13.75, 34.25)	14.60 (12.55, 29.70)	21.95 (18.85, 37.75)	0.236
Lactates (mmol·L^{-1})	1.10 (0.90, 1.40)	1.05 (0.90, 1.37)	1.10 (0.95, 1.40)	0.570
Lactate dehydrogenase (U·L^{-1})	603.50 (473.75, 727.50)	579.50 (422.50, 681.50)	640.00 (497.50, 727.50)	0.487
Total lymphocytes (10^9·L^{-1})	1.15 (0.89, 1.66)	1.53 (1.22, 1.81)	0.64 (0.54, 0.74)	0.064
Procalcitonin (μg L^{-1})	0.38 (0.19, 0.91)	0.24 (0.10, 0.61)	1.00 (0.28, 2.31)	0.023
Platelets (10^9·L^{-1})	327 (256, 376)	345 (266, 383)	317 (260, 355)	0.603
Eosinophilic polynuclear (10^9·L^{-1})	0.11 (0.00, 0.2)	0.02 (0.00, 0.1)	0.14 (0.00, 0.25)	0.133
Alpha TNF (pg·L^{-1})	17.30 (0.00, 29.45)	15.60 (0.00, 25.20)	20.60 (0.00, 31.12)	0.494

Values are expressed as median (interquartile ranges), absolute value (percentages); CRP (C-reactive protein)

Table S3. Biological values on ICU discharge

	Overall (n = 16)	Non-AKI (n = 7)	AKI (n = 9)	p-Value
**Urine Tests**				
Alpha1-microglobulin (mg·L^{-1})	119.00 (74.50, 193.50)	84.00 (42.50, 118.50)	129.00 (115.00, 382.00)	0.125
Urine IgG (mg·L^{-1})	17.15 (10.05, 24.75)	15.20 (7.15, 20.70)	22.30 (14.20, 24.90)	0.266
Urine microalbumin (g·L^{-1})	0.03 (0.02, 0.05)	0.02 (0.01, 0.03)	0.04 (0.02, 0.09)	0.186
Urine protein (g·L^{-1})	0.27 (0.11, 0.45)	0.27 (0.18, 0.33)	0.30 (0.11, 0.73)	0.522
Urine retinol binding protein (mg·L^{-1})	0.69 (0.00, 9.21)	0.00 (0.00, 2.51)	1.98 (0.00, 17.82)	0.329
Urine creatinine (mmol·L^{-1})	4.12 (2.17, 5.48)	4.65 (3.46, 5.42)	3.21 (2.02, 5.62)	0.699
Urine urea (mmol·L^{-1})	200.30 (143.80, 275.75)	200.30 (169.80, 243.30)	226.95 (130.38, 294.50)	0.643
**Blood Tests**				
Serum creatinine (µmol·L^{-1})	63.00 (41.50, 101.25)	46.00 (38.50, 77.00)	64.00 (42.00, 119.00)	0.223
Serum urea (mmol·L^{-1})	9.90 (6.22, 15.80)	6.50 (5.55, 9.50)	14.10 (8.60, 17.90)	0.138
CRP (mg·L^{-1})	126.20 (47.42, 217.50)	61.40 (30.70, 143.20)	153.00 (99.40, 215.00)	0.456
Ferritine (µg·L^{-1})	1130.00 (1093.00, 1167.00)	NA (NA, NA)	1130.00 (1093.00, 1167.00)	NA
Fibrinogen (g·L^{-1})	7.12 (5.86, 9.02)	8.95 (7.27, 9.09)	6.89 (5.94, 7.36)	0.655
Leucocytes (10^{9}·L^{-1})	11.56 (8.23, 13.76)	10.93 (9.25, 12.77)	12.98 (8.27, 16.43)	0.711
Interleukin 1 beta (pg·L^{-1})	0.00 (0.00, 0.00)	NA (NA, NA)	0.00 (0.00, 0.00)	NA
Interleukin 6 (pg·L^{-1})	230.65 (211.57, 249.73)	NA (NA, NA)	230.65 (211.57, 249.73)	NA
Interleukin 10 (pg·L^{-1})	38.80 (38.80, 38.80)	NA (NA, NA)	38.80 (38.80, 38.80)	NA
Lactates (mmol·L^{-1})	1.05 (0.80, 1.33)	1.10 (0.90, 1.35)	0.90 (0.70, 1.20)	0.366
Lactate dehydrogenase (U·L^{-1})	451.00 (386.00, 516.00)	NA (NA, NA)	451.00 (386.00, 516.00)	NA
Procalcitonin (µg·L^{-1})	0.43 (0.22, 0.62)	0.21 (0.10, 0.29)	0.66 (0.52, 0.78)	0.004
Platelets (10^{9}·L^{-1})	357 (284.75, 447)	443 (276, 462)	343 (285, 373)	0.560
Eosinophilic polynuclear (10^{9}·L^{-1})	0.22 (0.17, 0.29)	0.26 (0.22, 0.27)	0.2 (0.16, 0.38)	0.855
Alpha TNF (pg·L^{-1})	0.00 (0.00, 0.00)	NA (NA, NA)	0.00 (0.00, 0.00)	NA

Values are expressed as median (interquartile ranges), absolute value (percentages); CRP (C-reactive protein).

Table S4. In ICU and hospital prognosis.

	Overall (n = 42)	Non-AKI (n = 18)	AKI (n = 24)	p-Value
**In ICU Stay**				
Creatinine on ICU discharge (μmol·L^{-1})	68.50 (49.75, 127.50)	52.50 (43.00, 62.75)	101.00 (61.75, 230.75)	0.003
Creatinine clearance on ICU discharge (mL·min^{-1})	104.18 (49.94, 149.52)	138.97 (120.49, 171.70)	64.49 (25.16, 117.54)	0.002
Creatinine clearance < 60 mL·min^{-1}on ICU discharge (%)	11 (26.2)	0 (0.0)	11 (45.8)	0.001
Days between ICU admission and mechanical ventilation	0.00 (0.00, 0.50)	0.00 (0.00, 1.00)	0.00 (0.00, 0.00)	0.397
Days of mechanical ventilation	19.00 (11.00, 28.00)	17.00 (6.25, 23.75)	22.00 (12.00, 34.00)	0.173
LOS in ICU (days)	19.50 (14.00, 33.25)	20.00 (13.75, 30.75)	19.50 (15.50, 36.25)	0.507
**In Hospital Stay**				
Creatinine on hospital discharge (μmol.L-1)	73.00 (58.00, 162.00)	60.00 (53.00, 72.00)	161.50 (75.00, 219.50)	0.005
Creatinine clearance on hospital discharge (ml.min^{-1})	93.07 (37.91, 122.89)	121.04 (97.95, 134.73)	37.93 (27.10, 94.40)	0.005
Creatinine clearance < 60 ml.min^{-1} on hospital discharge (%)	9 (21)	0 (0.0)	9 (37.5)	0.001
LOS in hospital (days)	30.00 (18.50, 46.50)	43.00 (24.00, 48.00)	26.00 (15.00, 41.50)	0.205

Values are expressed as median (interquartile ranges), absolute value (percentages); LOS (length of stay)
